# Laparoscopic Ovarian Metastasectomy During Intraperitoneal Administration of Paclitaxel Chemotherapy for Gastric Cancer: A Report of Six Cases

**DOI:** 10.1155/cris/4451406

**Published:** 2026-07-26

**Authors:** Takao Kawai, Yoko Matsumoto, Nagiko Yoshida, Hana Yoshida, Mao Kamitani, Okikaze Kato, Saki Tanimoto, Gaku Kurishita, Tomomi Ishino, Akio Hidemura, Hideyuki Kagawa

**Affiliations:** ^1^ Department of Obstetrics and Gynecology, Kanto Rosai Hospital, 1–1 Kizukisumiyoshi-cho Nakahara-ku, Kawasaki 211–8510, Kanagawa, Japan, rofuku.go.jp; ^2^ Department of Surgery, Kanto Rosai Hospital, 1–1 Kizukisumiyoshi-cho Nakahara-ku, Kawasaki 211–8510, Kanagawa, Japan, rofuku.go.jp

**Keywords:** advanced gastric cancers, intraperitoneal paclitaxel, laparoscopy, metastatic tumors of the ovary

## Abstract

Intraperitoneal (IP) paclitaxel therapy with intravenous paclitaxel plus S‐1 is a novel treatment for advanced gastric cancer with peritoneal dissemination; however, its effectiveness against ovarian metastases remains limited. In such cases, ovarian metastasectomy may be considered as an alternative to chemotherapy, but its safety and efficacy are unclear. We reported the six cases of laparoscopic ovarian metastasectomy for gastric cancer during IP and intravenous paclitaxel plus S‐1 therapy. The median patient age was 40.5 years, and ovarian metastases were metachronous in five cases. All six surgeries were completed successfully without complications, adhesions, or conversion to open surgery. The median interval between ovarian metastasectomy and resumption of IP paclitaxel chemotherapy was 11.5 days. Three patients had a large amount of ascites before surgery, but the ascites decreased after the procedure. Four patients died from cancer, with a median postoperative survival of 18.5 months, excluding two patients who remained alive. Laparoscopic ovarian metastasectomy was feasible when peritoneal dissemination was well controlled by IP paclitaxel, and there were no strong adhesions around the ovarian metastases, which were presumed to have spread via lymphatic or hematogenous routes. Furthermore, ovarian metastasectomy reduced postoperative ascites formation, and the minimally invasive approach enabled the early resumption of IP paclitaxel therapy after a short treatment suspension. As a limitation, it was difficult to evaluate improvement in oncologic outcomes because of strong selection bias. In conclusion, laparoscopic ovarian metastasectomy is a safe and viable option for selective patients; however, further research is needed to confirm its long‐term benefits.

## 1. Introduction

Ovarian metastasis accounts for ~10% of all ovarian cancers [1], with 20%–34% of these cases originating from gastric cancer [2, 3]. Although ovarian metastasis from gastric cancer is generally associated with poor prognosis [2], surgical resection of ovarian metastases may improve outcomes in specific patients with metachronous tumors and good performance status [1, 4, 5], but the evidence was limited. In addition, because gastric cancer recurrence frequently involves peritoneal dissemination [6], laparoscopic surgical resection can be challenging owing to the potential for significant peritoneal adhesions. This has raised concerns about the safety of laparoscopic ovarian metastasectomy, highlighting the importance of controlling peritoneal dissemination before surgery.

At our hospital, peritoneal dissemination of gastric cancer is managed with a novel treatment regimen of intraperitoneal (IP) and intravenous paclitaxel plus S‐1 (IP chemotherapy) [7]. IP chemotherapy is considered when distant disease was primarily peritoneal dissemination and no metastases other than the ovary were present, after obtaining sufficient informed consent. This treatment strategy differed from the Japanese gastric cancer treatment guidelines, but IP chemotherapy was considered a good option for patients whose distant metastases were mainly peritoneal, as previous clinical studies, such as the PHOENIX‐GC trial [7], suggested that the 3‐year survival rate of the patients with IP chemotherapy was higher than standard S‐1 plus cisplatin therapy, with a relatively larger proportion of patients achieving long‐term survival, despite the lack of a statistically significant difference in overall survival.

Ovarian metastases from gastric cancer during IP chemotherapy are surgically resected by proficient gynecologists at our facility. The indication for resection is determined on a case‐by‐case basis after consultation between the surgeon and the gynecologist when the patient’s performance status is 0–1, peritoneal dissemination is under control, and the metastatic ovarian tumor is resectable. The primary purpose of surgery is to remove progressive ovarian metastasis, expected to have a favorable oncologic prognosis. This was because the ovaries alone showed a poor response to IP chemotherapy. The additional purpose of surgery was to improve quality of life by reducing abdominal mass sensation and ascites and prevent ovarian torsion.

Minimally invasive surgery is performed for ovarian metastasectomy if the ovaries are not too large to be stored in the surgical retrieval bag. The determination of suitability for laparoscopic surgery at our facility is not based on a strict size criterion, although 10 cm is considered a benchmark. The indication for laparoscopic surgery was determined based on a comprehensive evaluation of multiple factors, including ovarian size and the absence of significant adhesions. Adhesions were assessed by preoperative pelvic examination and computed tomography to confirm the absence of adhesions that could make insertion of the first port difficult.

We encountered six successful cases of laparoscopic ovarian metastasectomy for gastric cancer with peritoneal dissemination. This report presents these cases, with a focus on the safety and efficacy of laparoscopic ovarian metastasectomy. We evaluated safety based on the presence or absence of intraoperative complications, open abdominal conversion, and tumor rupture and evaluated efficacy based on patient prognosis after surgery, the controllability of ascites, and whether chemotherapy could be resumed immediately after surgery.

## 2. Case Series

**Figure 1:**
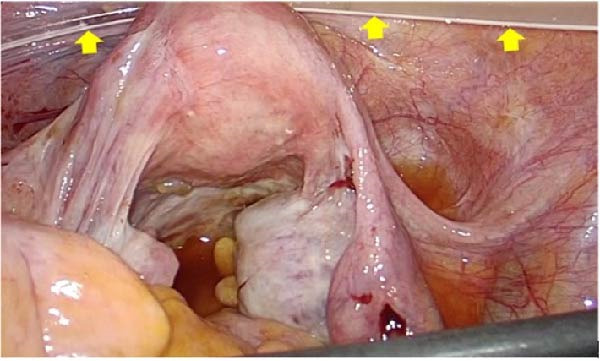
Operative view of the bilateral ovary from Case 2. No adhesions were observed around the bilateral ovaries. Yellow arrows indicate the tube for intraperitoneal paclitaxel injection.

**Figure 2:**
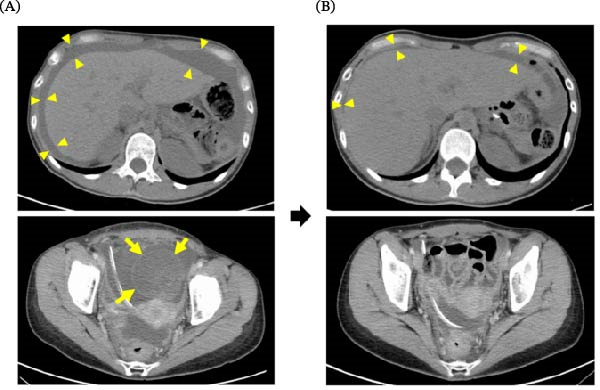
Perioperative computed tomography images of the abdomen and pelvis in Case 1. (A) The findings 2 months before surgery. (B) The findings 1 month after surgery. The yellow arrowhead indicates a marked reduction in perihepatic ascites. The yellow arrow indicates the enlarged right ovary.

**Figure 3:**
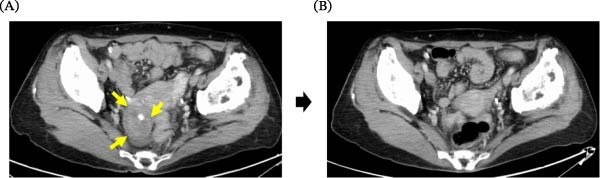
Perioperative computed tomography images of the pelvis in Case 2. (A) The findings 2 weeks before surgery. (B) The findings 2 months after surgery. The yellow arrow indicates the enlarged right ovary.

**Figure 4:**
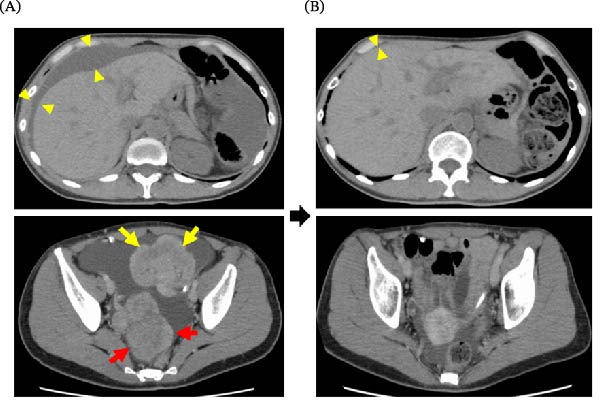
Perioperative computed tomography images of the abdomen and pelvis in Case 3. (A) The findings 1 month before surgery. (B) The findings 2 months after surgery. The yellow arrowhead indicates a marked reduction in perihepatic ascites. The yellow arrow indicates the enlarged left ovary, and the red arrow indicates the enlarged right ovary.

**Figure 5:**
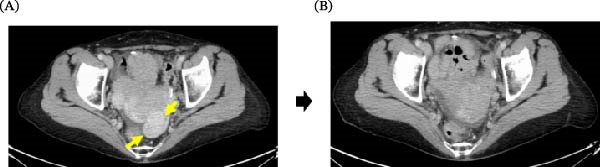
Perioperative computed tomography images of the pelvis in Case 4. (A) The findings 1 month before surgery. (B) The findings 2 months after surgery. The yellow arrow indicates the enlarged left ovary.

**Figure 6:**
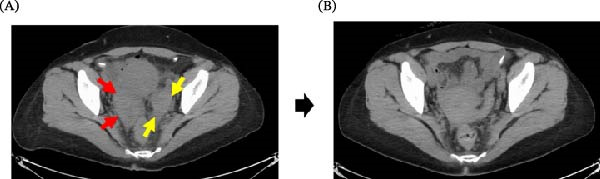
Perioperative computed tomography images of the pelvis in Case 5. (A) The findings 1 week before surgery. (B) The findings 1 month after surgery. The yellow arrow indicates the enlarged left ovary, and the red arrow indicates the enlarged right ovary.

**Figure 7:**
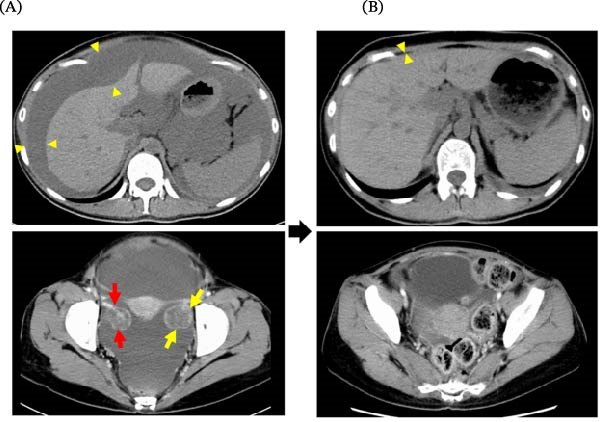
Perioperative computed tomography images of the abdomen and pelvis in Case 6. (A) The findings 2 weeks before surgery. (B) The findings 2 months after surgery. The yellow arrowhead indicates a marked reduction in perihepatic ascites. The yellow arrow indicates the enlarged left ovary, and the red arrow indicates the enlarged right ovary.

From January 2018 to March 2024, six cases of laparoscopic ovarian metastasectomy for gastric cancer with peritoneal dissemination were identified. The surgical methods, safety, operative findings, and effectiveness of the procedures are summarized in Table 1. A representative intraoperative image of the bilateral ovaries in Case 2 is shown in Figure 1. Figures 2–7 show representative images of ovarian metastases in all cases, as well as changes in the amount of ascites before and after surgery in Cases 1, 3, and 6. The representative surgical procedures for all cases were as follows: four trocars were placed in a diamond‐shaped arrangement in the lower abdomen. After opening the retroperitoneum, the ureters were identified. The infundibulopelvic ligaments were then transected, the broad ligaments were resected, and the ovarian ligaments were transected. The procedure was performed identically on both sides, and both bilateral adnexa were removed. Specimens were placed into a surgical retrieval bag and removed through a small umbilical incision while cutting the specimen. After peritoneal lavage and confirming hemostasis, the operation was completed. There was no strong adhesion, rupture during surgery, or operational complications in any of the cases.

**Table 1:** Summary of six cases.

Case number	Case 1	Case 2	Case 3	Case 4	Case 5	Case 6
Age at surgery (years)	37	62	41	49	40	35
Metastasis timing	Metachronous	Metachronous	Metachronous	Metachronous	Metachronous	Synchronous
Period from diagnosis of gastric cancer to metastasectomy (months)	33	27	16	18	15	5
Size of ovary (diameter)	Right 10 cm	Right 5 cm	Right 7 cm	Right not swell	Right 5 cm	Right 7.5 cm
	Left 4 cm	Left atrohpic	Left 10 cm	Left 4 cm	Left 4 cm	Left 6 cm
Adhesion around ovary	Filmy adhesion	(−)^a^	(−)	(−)	(−)	(−)
Blood loss during surgery (ml)	150 (including ascites)	0	0	0	200 (with colostomy augmentation)	0
The operative time (minutes)	100	129	174	90	153	149
Hospital stay around surgery (days)	6	6	6	6	4	16 (Including purposes other than surgery)
Rupture of the ovarian capsule	(−)	(−)	(+) (before surgery)	(−)	(−)	(−)
Complication	(−)	(−)	(−)	(−)	(−)	(−)
Pathology	Poorly differentiated adenocarcinoma, bilateral ovary	Signet ring cell carcinoma, bilateral ovary	Poorly differentiated adenocarcinoma, bilateral ovary	Poorly differentiated adenocarcinoma and signet ring cell carcinoma, bilateral ovary	Poorly differentiated adenocarcinoma and signet ring cell carcinoma, bilateral ovary	Signet ring cell carcinoma, bilateral ovary
Period from surgery to chemotherapy (days)	6	10	13	13	19	4
Ascites	↓^b^	(−)	↓	(−)	(−)	↓
Present status	DOD	DOD	DOD	DOD	AWD	AWD
Prognosis after surgery (months)	14	25	12	23	9	8
Prognosis after initiation of IP chemotherapy (months)	18	28	27	39	22	8

Abbreviations: AWD, alive with disease; DOD, dead of disease.

^a^(−) means not observed or detected.

^b^↓ means decreased.

All cases were treated after approval by our institutional ethics committee. The treatment regimen for IP paclitaxel consisted of paclitaxel 20 mg/m^2^ intraperitoneally and paclitaxel 50 mg/m^2^ intravenously on days 1 and 8, and S‐1 80 mg/m^2^ daily from day 1–14 in a 3‐week cycle, which was the same as in the PHOENIX‐GC trial [7]. IP chemotherapy was not covered by health insurance at that time, so it was administered at the patients’ own expense at our facility. No other insurance‐covered treatments, such as surgery or other chemotherapy, were performed concurrently with the IP therapy to avoid mixed billing. Informed consent was obtained from all patients using an opt‐out method, and patient anonymity was preserved in the present study. Ethical approval was obtained from our Institutional Ethics Committee (Number 2024‐3).

### 2.1. Case 1

A 37‐year‐old woman with an unremarkable medical history was diagnosed with resectable gastric cancer. The patient underwent a total gastrectomy and splenectomy, followed by adjuvant chemotherapy. One year after chemotherapy completion, the patient experienced recurrence with peritoneal dissemination and ovarian metastasis. Human epidermal growth factor receptor 2 (HER2) testing was negative, and IP chemotherapy was selected after obtaining sufficient informed consent. An IP port was placed, and five cycles of IP chemotherapy were administered. The disseminated disease remained stable; however, the ovaries continued to enlarge, prompting laparoscopic salpingo‐oophorectomy, expected to have a favorable oncologic prognosis and reduce ascites. The operative time was 100 min, and the length of hospital stay around surgery was 6 days. There was no perioperative complication. Six days after surgery, IP chemotherapy was resumed. One month after surgery, the large volume of ascites extending beyond the pelvic cavity was reduced to a small amount localized within the pelvis (Figure 2). The patient continued receiving IP chemotherapy for 11 months. However, after starting the third‐line regimen, she developed a cerebral infarction and died 2 weeks later, specifically 1 year and 2 months after ovarian metastasectomy.

### 2.2. Case 2

A 62‐year‐old woman with a history of breast cancer treatment was diagnosed with resectable gastric cancer. The patient underwent a total gastrectomy and splenectomy without adjuvant chemotherapy. One year and 10 months later, the patient experienced a recurrence with peritoneal dissemination. HER2 testing was negative, and IP chemotherapy was selected after obtaining sufficient informed consent. An IP port was placed, and five cycles of IP chemotherapy were administered. The disseminated disease stabilized; however, ovarian metastases appeared (Figure 3), leading to laparoscopic salpingo‐oophorectomy, expected to have a favorable oncologic prognosis. The operative time was 129 min, and the length of hospital stay around surgery was 6 days. There was no perioperative complication. Ten days after the operation, IP chemotherapy was resumed. Nine months after surgery, the patient continued IP chemotherapy. However, the patient developed ileus as a result of the cancerous peritonitis, which required conservative management. Despite receiving second‐line chemotherapy, her cancer progressed rapidly, leading to loss of consciousness, and she died 2 years and 1 month after ovarian metastasectomy.

### 2.3. Case 3

A 41‐year‐old woman with an unremarkable medical history was diagnosed with resectable gastric cancer. The patient underwent total gastrectomy and splenectomy despite peritoneal dissemination during surgery. HER2 testing was negative, and IP chemotherapy was initiated after obtaining sufficient informed consent. After 19 cycles of adjuvant IP chemotherapy, the patient’s ovaries gradually enlarged, and she experienced abdominal bloating, prompting laparoscopic salpingo‐oophorectomy, expected to have a favorable oncologic prognosis and reduce ascites. The operative time was 174 min, and the length of hospital stay around surgery was 6 days. There was no perioperative complication. Thirteen days after surgery, IP chemotherapy was resumed. The large volume of ascites extending beyond the pelvic cavity was reduced to a small amount localized within the pelvis 2 months after surgery (Figure 4). The patient continued to receive IP chemotherapy for 7 months. However, she subsequently developed obstructive jaundice, pneumonia, and a cervical bone compression fracture, which led to cancer progression and death 1 year after ovarian metastasectomy.

### 2.4. Case 4

A 49‐year‐old woman with no significant medical history was diagnosed with gastric cancer. Diagnostic laparoscopy revealed gastric cancer with peritoneal dissemination that was initially deemed unresectable. HER2 testing was negative, and IP chemotherapy was selected after obtaining sufficient informed consent. After eight cycles of IP chemotherapy, peritoneal dissemination was controlled, allowing for a total gastrectomy. Following surgery, 11 additional cycles of IP chemotherapy were administered. Ovarian metastases developed during this period (Figure 5), necessitating laparoscopic salpingo‐oophorectomy, expected to have a favorable oncologic prognosis. The operative time was 90 min, and the length of hospital stay around surgery was 6 days. There was no perioperative complication. Thirteen days after surgery, IP chemotherapy was resumed. The patient continued IP chemotherapy for 13 months after surgery. However, the patient developed an intestinal obstruction, necessitating a colostomy. During second‐line chemotherapy treatments, the patient died of septic shock resulting from a central venous port infection 1 year and 11 months after ovarian metastasectomy.

### 2.5. Case 5

A 40‐year‐old woman with no significant medical history was diagnosed with unresectable gastric cancer with peritoneal dissemination. HER2 testing was negative, and IP chemotherapy was selected after obtaining sufficient informed consent. An IP port was placed, and five cycles of IP chemotherapy were administered. Subsequently, the patient developed defecation difficulties, and colonoscopy revealed a lower gastrointestinal obstruction, indicating disease progression. A second‐line regimen consisting of folinic acid, fluorouracil, and oxaliplatin was initiated; however, the patient continued to receive IP paclitaxel monotherapy every 2 weeks at her request. Four months after the initiation of the second‐line regimen, the patient’s defecation symptoms did not improve, and she underwent a colostomy. At that time, ovarian metastases were detected (Figure 6); therefore, ovarian metastasectomy, expected to have a favorable oncologic prognosis, was performed simultaneously during colostomy. The operative time was 153 min, and the length of hospital stay around surgery was 4 days. There was no perioperative complication. Nineteen days after surgery, IP paclitaxel monotherapy was resumed. Nine months postoperatively, the patient was alive as of the writing of this paper and continued treatment with IP paclitaxel monotherapy despite switching to a third‐line regimen.

### 2.6. Case 6

A 35‐year‐old woman with no significant medical history was diagnosed with unresectable gastric cancer with peritoneal dissemination. Initially, the patient received a first‐line regimen of nivolumab, folinic acid, fluorouracil, and oxaliplatin at another facility. However, her ascites remained uncontrolled, prompting four cycles of hyperthermic IP chemotherapy, which had little effect. She was referred to our hospital, where HER2 testing was negative, and microsatellite instability testing indicated microsatellite stability. As a result, IP chemotherapy was selected after obtaining sufficient informed consent. At this time, ovarian metastases appeared, and laparoscopic metastasectomy expected to have a favorable oncologic prognosis and reduce ascites was performed concurrently with IP port placement. The operative time was 149 min, and the length of hospital stay around surgery was 16 days, including the hospitalization period for surgical preparation and chemotherapy stay. There was no perioperative complication. Four days after surgery, IP chemotherapy started. The large volume of ascites extending beyond the pelvic cavity was reduced to a small amount localized within the pelvis 2 months after surgery (Figure 7). Eight months postoperatively, the patient continued IP chemotherapy and was alive as of the writing of this paper.

## 3. Discussion

In summary, the median age at surgery was 40.5 years among the six cases. Metastases were synchronous in five cases and metachronous in one case. The median period from the diagnosis of gastric cancer to metastasectomy was 17 months. The size of the ovaries varied from normal size to 10 cm in diameter. Five cases showed no adhesion, and one case had only a filmy adhesion. In four cases, intraoperative blood loss was minimal; in one case, it was 150 mL, including ascites; and in another case, it was 200 mL, including blood loss from colostomy augmentation. The median operative time was 139 min, and the median length of hospital stay around surgery was 6 days. Five cases showed no collapse, and one case still had collapse. No cases had collapse due to the surgical procedure. There were no complications of the surgery in any of the cases. The ovarian pathology was poorly differentiated adenocarcinoma in two cases, signet ring cell carcinoma in two cases, and both poorly differentiated adenocarcinoma and signet ring cell carcinoma in two cases. The median period from surgery to chemotherapy was 11.5 days. In three cases, major ascites that were not localized to the pelvic cavity were present, but the amount of ascites apparently decreased after surgery. In the other three cases, no major ascites were present. Four of the patients had already died, but two were still alive at the time of writing this paper. The median survival after surgery was 13 months, and the median survival after the initiation of IP chemotherapy was 24.5 months.

In these cases, minimally invasive surgery for ovarian metastases was successfully performed, with all patients experiencing no complications, minimal blood loss, short intervals between surgery and chemotherapy, and no need for conversion to open surgery. In five of the six cases, the ovaries were resected without rupture, and no patients exhibited tight adhesions around the ovaries. Our findings suggest that laparoscopic approaches may be feasible and beneficial in carefully selected patients when peritoneal dissemination was well controlled by IP paclitaxel, and there were no strong adhesions around the ovarian metastases.

Oncologic outcomes are difficult to evaluate appropriately because of the strong selection bias and because the treatment approach differed from guideline recommendations. Despite these limitations, comparison with previous reports may still be informative. The median overall survival from the initiation of IP chemotherapy was 24.5 months, which exceeds the median survival previously reported for patients treated with chemotherapy alone, such as IP chemotherapy (17.7 months) or standard chemotherapy with S‐1 plus cisplatin (15.2 months) [7]. While this result cannot establish a survival benefit due to the small sample size and potential selection bias, like healthier patients selected for surgery, the improved control of ascites may contribute to better compliance with chemotherapy, potentially enhancing survival indirectly. Further studies are warranted to clarify the oncologic impact of metastasectomy.

For advanced or recurrent gastric cancer, treatment recommendations in the national guidelines [8] vary according to molecular biomarkers such as HER2, CLDN18, PD‐L1, and MSI. At the time these cases were treated, however, only HER2 testing was performed because of limitations in insurance coverage, and all cases were HER2‐negative. According to the guidelines at that time, the standard treatment was S‐1 plus cisplatin. Nevertheless, after obtaining detailed informed consent, we decided to administer IP chemotherapy. This decision was based on the findings of the PHOENIX‐GC trial: although no significant difference in overall survival was demonstrated in the overall population, subgroup analysis suggested potential benefit in patients with ascites extending beyond the pelvic cavity, and the 3‐year survival rate was 21.9%, indicating a relatively high proportion of long‐term survivors. We therefore considered that, with appropriate patient selection, there may be a subgroup of patients who could benefit from this treatment. Although surgical resection of ovarian metastases is not recommended in the guidelines and systemic chemotherapy remains the principal treatment modality, we considered that surgery may be an option in selected patients whose disease shows no progression except for ovarian metastases, with peritoneal dissemination controlled by IP chemotherapy.

The other benefit of the surgery was to improve quality of life by reducing abdominal mass sensation and ascites and to prevent ovarian torsion. In three cases, the ascites volume significantly decreased following surgery. Notably, in Cases 1 and 3, this improvement occurred without changes to chemotherapy regimens, and postoperative ascites control has been maintained until death or the present day in all six cases. This phenomenon is consistent with pseudo‐Meigs syndrome, characterized by the resolution of ascites and pleural effusion after bilateral oophorectomy [9]. Krukenberg tumors of gastric origin have been reported as a cause of this syndrome, possibly due to peritoneal irritation from solid ovarian tumors, lymphatic obstruction, stromal edema, or increased vascular permeability mediated by inflammatory cytokines and VEGF [10].

In terms of safety in minimally invasive surgery, several factors may contribute to the success of minimally invasive surgery for ovarian metastasis of gastric cancer during IP chemotherapy. First, ovarian metastases from gastric cancer primarily spread via lymphatic or hematogenous routes rather than transcoelomic dissemination [1]. Consequently, adhesions around ovarian metastases tend to be less severe. Second, the progression of peritoneal dissemination in these cases was controlled by IP paclitaxel, reducing the severity of adhesions between the intestinal tract and abdominal wall thanks to the antiadhesive effect of paclitaxel, which facilitated port placement and maintained a clear operative field [11–13]. Third, the primary surgical site for gastric cancer is the upper abdomen. Therefore, postoperative adhesions are mainly located in the upper abdomen, with minimal involvement of the pelvis.

In addition to these six cases, three open ovarian metastasectomies during IP chemotherapy were performed during the same period (January 2018 to March 2024). The reasons for choosing open surgery in these cases included large ovarian size (one ovary measured 17 cm in diameter), the presence of abdominal wall metastasis that needed to be removed simultaneously, and the need for emergency surgery because of a suspected strangulated ileus. Regarding bleeding, two of the three cases undergoing open surgery experienced intraoperative blood loss ranging from 200 to 240 mL. The remaining case required a blood transfusion, although the exact amount of blood loss was not recorded. The operative time was 244 min, including surgical procedures other than ovarian metastasectomy, and the median length of hospital stay was 44 days, including hospitalization for purposes other than surgery. Chemotherapy was resumed 14 days after open surgery in one case; the other two cases did not resume chemotherapy but proceeded to palliative care. In these patients, ovarian metastasectomy was performed concurrently with palliative colostomy. It was suggested that minimally invasive surgery may result in less bleeding and a shorter time to chemotherapy resumption. However, because of the small sample size, further validation is required. Generally, minimally invasive surgery offers advantages such as reduced postoperative pain and shorter hospitalization periods. However, it has disadvantages, including difficulty in its application to cases with adhesions and the need for surgeons to have specialized training and sufficient experience in minimally invasive techniques [14].

To our knowledge, this is the first case series describing the safety of laparoscopic ovarian metastasectomy for gastric cancer with peritoneal dissemination during IP chemotherapy. However, this study includes a small number of cases and is subject to selection bias as it involved patients only with good performance status and controlled peritoneal dissemination under IP chemotherapy. Furthermore, the favorable outcomes observed here occurred under IP paclitaxel‐based chemotherapy, which is not routinely used in all gastric cancer cases. Therefore, these results may not be generalizable to all patients with ovarian metastases from gastric cancer, particularly those not receiving IP chemotherapy. Additionally, we were not able to objectively assess the patients’ symptoms of abdominal bloating, which weakened the evidence supporting the palliative purpose of the surgery.

In conclusion, we report six cases of successful and safe laparoscopic ovarian metastasectomy for gastric cancer with peritoneal dissemination during IP chemotherapy. Although the management of ovarian metastases in this context presents challenges, our findings suggest that laparoscopic approaches may be feasible and beneficial in carefully selected patients. Further research is necessary to better define the criteria for surgical intervention and the potential long‐term benefits of ovarian metastasectomy in this patient population.

## Funding

The authors received no specific funding for this work.

## Ethics Statement

Informed consent was obtained from all patients using an opt‐out method, and patient anonymity was preserved in the present study. Ethical approval was obtained from our Research Ethics Committee (Number 2024‐3).

## Consent

Written informed consent could not be obtained because the patients were deceased and contact with their families was not feasible. Therefore, all data were appropriately anonymized, and an opt‐out method was also employed.

## Conflicts of Interest

The authors declare no conflicts of interest.

## Data Availability

The data that support the findings of this study are available upon request from the corresponding author. The data are not publicly available due to privacy or ethical restrictions.
